# The use of plant lectins to regulate H1N1 influenza A virus receptor binding activity

**DOI:** 10.1371/journal.pone.0195525

**Published:** 2018-04-09

**Authors:** Nicolette Lee, Alexey M. Khalenkov, Vladimir Y. Lugovtsev, Derek D. Ireland, Anastasia P. Samsonova, Nicolai V. Bovin, Raymond P. Donnelly, Natalia A. Ilyushina

**Affiliations:** 1 Division of Biotechnology Research and Review II, Center for Drug Evaluation and Research, U.S. Food and Drug Administration, Silver Spring, Maryland, United States of America; 2 Division of Plasma Protein Therapeutics, Center for Biologics Evaluation and Research, U.S. Food and Drug Administration, Silver Spring, Maryland, United States of America; 3 Division of Viral Products, Center for Biologics Evaluation and Research, U.S. Food and Drug Administration, Silver Spring, Maryland, United States of America; 4 Carbohydrate Chemistry Laboratory, Shemyakin Institute of Bioorganic Chemistry, Russian Academy of Sciences, Moscow, Russia; University of South Dakota, UNITED STATES

## Abstract

We applied an *in vitro* selection approach using two different plant lectins that bind to α2,3- or α2,6-linked sialic acids to determine which genetic changes of the A/California/04/09 (H1N1) virus alter hemagglutinin (HA) receptor binding toward α2,3- or α2,6-linked glycans. Consecutive passages of the A/California/04/09 virus with or without lectins in human lung epithelial Calu-3 cells led to development of three HA1 amino acid substitutions, N129D, G155E, and S183P, and one mutation in the neuraminidase (NA), G201E. The S183P mutation significantly increased binding to several α2,6 SA-linked glycans, including YDS, 6′SL(N), and 6-Su-6′SLN, compared to the wild-type virus (↑3.6-fold, *P* < 0.05). Two other HA1 mutations, N129D and G155E, were sufficient to significantly increase binding to α2,6-linked glycans, 6′SLN and 6-Su-6′SLN, compared to S183P (↑4.1-fold, *P* < 0.05). These HA1 mutations also increased binding affinity for 3′SLN glycan compared to the wild-type virus as measured by Biacore surface plasmon resonance method. In addition, the HA1 N129D and HA1 G155E substitutions were identified as antigenic mutations. Furthermore, the G201E mutation in NA reduced the NA enzyme activity (↓2.3-fold). These findings demonstrate that the A/California/04/09 (H1N1) virus can acquire enhanced receptor affinity for both α2,3- and α2,6-linked sialic receptors under lectin-induced selective pressure. Such changes in binding affinity are conferred by selection of beneficial HA1 mutations that affect receptor specificity, antigenicity, and/or functional compatibility with the NA protein.

## Introduction

Hemagglutinin (HA) and neuraminidase (NA), the surface glycoproteins of influenza virus, play vital roles in the virus life cycle. HA binds to glycan receptors on the host cell surface to initiate fusion of the cell and viral membranes; whereas, NA enzymatically cleaves sialic acids (SAs) from glycans to facilitate the release of budding progeny viruses [1,2]. Human influenza viruses preferentially recognize glycans with terminal α2,6-linked SAs, which are broadly distributed on epithelial cells of the human trachea. In contrast, avian influenza viruses specifically bind α2,3-linked SAs, which are present only in the alveoli in the lower human respiratory tract [3]. An optimal balance between HA receptor binding and NA receptor destroying is important for efficient viral replication in cultured cells, mice and humans [3–7]. However, mismatched pairs of HA and NA can be rescued by adaptive mutations in the HA, NA, or both proteins after several replication cycles [4,8–10].

In 2009, the emergence of a novel H1N1 influenza A virus originating from swine resulted in a pandemic [11]. This virus showed a functional balance between its HA and NA proteins [3], demonstrating coadaptation of both proteins. In particular, the first identified pandemic strain, A/California/04/09 (CA/04), exhibited both low HA avidity for α2,6 sialic receptors and weak NA enzymatic activity in catalyzing α2,6-linked glycans [3]. Four weeks later, a distinct virus, A/New York/06/09, was isolated, and this strain accumulated four amino acid substitutions in the HA and two mutations in the NA. These amino acid substitutions allowed A/New York/06/09 to achieve a new functional balance, with improved HA binding avidity and enhanced NA activity towards α2,6-linked SAs [3]. The mutations acquired by this virus are present in most descendants of the pandemic viruses subsequently isolated from humans, suggesting that viruses carrying these mutations are fit for viral circulation in humans. In contrast, another strain isolated at the end of April 2009, A/Netherlands/602/09, showed improved HA binding for α2,6-linked SAs over CA/04 but greatly diminished NA activity, indicating an imbalanced HA/NA pair. The mismatch of the viral surface proteins put the A/Netherlands/602/09 virus in an evolutionary dead end because the mutations that impaired the HA/NA balance in this virus are not present in any other H1N1 pandemic strains, suggesting that it might not be fit for productive replication in humans [3].

Viral evolution is driven by natural selection of random mutations, which are generated rapidly due to the poor proofreading mechanism of the influenza virus RNA polymerase. Because of the randomness of the process, most mutants have impaired fitness and fail to propagate further. However, those mutations that provide selective advantages in the process of virus replication continue to accumulate in the viral genome, driving influenza virus evolution. It is a significant scientific challenge to predict all structural and functional changes that may arise and improve influenza virus replication in humans. In this study, we investigated whether the CA/04 virus could be further adapted by the fine tuning of HA receptor recognition for better fitness in human airway epithelium and whether it could result in the selection of variants with enhanced growth properties and more balanced HA/NA activities. We used two plant lectins, the *Maackia amurensis* agglutinin (MAA) and the *Sambucus nigra* agglutinin (SNA) that recognize α2,3- and α2,6-linked SAs, to drive *in vitro* selection of the 2009 pandemic H1N1 virus with predominant binding to α2,6- or α2,3-linked glycans. We hypothesized that H1N1 mutants selected by consecutive passage in the presence of lectins in a human lung epithelial cell line, Calu-3, might help to identify structural changes, which may occur in influenza virion when primary SA-containing host cellular receptors are masked and are not easily available for virus attachment.

## Materials and methods

### Cells, viruses, and lectins

The Madin-Darby canine kidney (MDCK) cell line and the human lung epithelial cell line (Calu-3) were obtained from the American Type Culture Collection (Manassas, VA, USA) and maintained as described elsewhere [12].

Human influenza CA/04 virus was kindly provided by Dr. Robert G. Webster (St. Jude Children’s Research Hospital, Memphis, TN). Viruses’ stocks were prepared by one passage either in the allantoic cavities of 10 day-old embryonated chicken eggs or in MDCK cells for 48 h at 37°C, and aliquots were stored at −70°C until use. All experimental work was performed in a biosafety level-2 (BSL-2) laboratory approved for use of these strains by the U.S. Department of Agriculture and the U.S. Centers for Disease Control and Prevention.

MAA lectin with carbohydrate specificity towards α2,3-linked SAs and SNA lectin with carbohydrate specificity towards α2,6-linked SAs (according the vendor’s specifications) were obtained from EY Laboratories, Inc. (San Mateo, CA, USA).

### Infectivity of H1N1 influenza viruses

The infectivity of H1N1 viruses was determined by plaque assay [13] and the 50% tissue culture infective dose (TCID_50_) [7]. Briefly, confluent cultures of MDCK cells were incubated at 37°C for 1 h with 10-fold serial dilutions of each virus. The cells were then washed and overlaid with minimal essential medium (MEM) containing 0.3% bovine serum albumin (BSA), 0.9% Bacto agar, and 1 μg/ml l-(tosylamido-2-phenyl)ethylchloromethylketone (TPCK)-treated trypsin. After 3 days of incubation at 37°C, the cells were stained with 0.1% crystal violet in 10% formaldehyde solution, and the number of plaque-forming units (PFU) per milliliter and plaque size of any 10 plaques were determined using a Finescale magnifying comparator.

The TCID_50_ was determined in confluent monolayers of MDCK cells grown in 96-well plates and inoculated with serial virus dilutions (each dilution was added to five wells) in the presence of 1 μg/ml TPCK-treated trypsin. After 3 days, virus was titrated by hemagglutination assay, and virus titers were expressed as log_10_TCID_50_/ml by the endpoint method of Reed & Muench [14].

### Virus yield reduction assay

The extracellular virus yield reduction assay was performed as described previously in 24-well plates containing confluent Calu-3 cells grown on membrane supports (6.5-mm Transwell, Corning Inc., Corning, NY, USA) [12]. The concentrations of MAA or SNA lectins ranged from 1 to 1000 μg/ml and they were added to the apical compartment of Calu-3 cells for 2 h. After pretreatment, the cells were overlaid with 2× lectin-containing medium (100 μl/well), infected with influenza virus at a multiplicity of infection (MOI) of 0.1 PFU/cell, and incubated for 48 h at 37°C. Virus yields were determined as the number of PFU/ml in MDCK cells. The drug concentration that caused a 50% decrease in the PFU titer in comparison to control wells without lectin was defined as EC_50_. The results of two independent experiments were averaged.

### Viral replication kinetics

To determine multistep growth curves for each virus, Calu-3 cells were inoculated via the apical side with the H1N1 viruses at an MOI of 0.001 PFU/cell. After incubation for 1 h, the cells were washed and overlaid with MEM medium containing 0.3% BSA and 1 μg/ml TPCK-treated trypsin. The supernatants were collected at 6, 24, 48, and 72 h post-infection and stored at −70°C until titration.

### Virus sequence analysis

Viral RNAs were isolated from virus-containing cell culture fluid after passages in Calu-3 cells followed by plaque purification in MDCK cells by using RNeasy Minikits (Qiagen, Germantown, MD, USA). Samples were reverse transcribed and analyzed by PCR using universal primers specific for influenza gene segments as described previously [15]. Sequencing was performed by the Research Central Facility for Biotechnology Resources at the U.S. Food and Drug Administration, Silver Spring, MD. The DNA template was sequenced using rhodamine or dichlororhodamine (drhodamine) dye terminator cycle sequencing Ready Reaction Kits with AmpliTaq DNA Polymerase FS (PerkinElmer Applied Biosystems, Waltham, MA, USA) and synthetic oligonucleotides. All samples were analyzed in a Perkin-Elmer Applied Biosystems DNA sequencer (model 373 or 377). DNA sequences were completed and edited by using a Lasergene sequence analysis software package (DNASTAR).

### Hemagglutination inhibition (HI) test and microneutralization (MN) assay

HI test was performed with 0.5% chicken red blood cells by a standard method as described elsewhere [16]. We used ferret antiserum obtained against A/New Jersey/15/07 (H1N1), CA/04, A/California/07/09 (H1N1), and A/Tennessee/1-560/09 (H1N1); goat antiserum obtained against CA/04; and a panel of 10 mouse monoclonal antibodies (MAbs) to HAs of 4 H1 strains: 4 antibodies to CA/04, MAbs 28665, 28666, 28667, and 28668; 2 antibodies to A/South Carolina/1/1918 (H1N1), MAbs NR-13451 and NR-13455; 2 antibodies to A/swine/AR/2976/02 (H1N2), MAbs H5 and G7; and 2 antibodies to A/swine/NC/18161/02 (H1N1), MAbs E2 and H7.

Virus-neutralizing MN titers were determined by infection of MDCK cells and expressed as the reciprocal of the highest MAb dilution that neutralized 50% of 100 TCID_50_ of virus after incubation at 37°C for 72 h.

### Virus purification

Allantoic fluid was clarified by low-speed centrifugation. The virus was pelleted and then purified through 27% and 49% (w/v) sucrose cushions. Virus-containing bands were pelleted and stored in phosphate-buffered saline (PBS) at −70°C until use. HA and NA concentrations were determined by optical densitometry of the SDS-PAGE gel images (S1 Fig) and total protein content was determined by BCA protein assay (Pierce Biotechnology, Rockford, IL, USA).

### Receptor-binding assay

The affinity of each virus for biotinylated 3′- and 6′-sialylglycopolymers was measured in a direct binding assay as described previously [12,17]. Briefly, plates were pre-coated with each virus at 4°C for 16 h, followed by washing with 0.05% Tween 20 in PBS (PBS-T). After the addition of biotinylated sialylglycopolymer in PBS supplemented with 0.02% Tween 20, 0.02% BSA, and 3 mM oseltamivir carboxylate, plates were incubated at 4°C for 1 h. Plates were then washed with cold PBS-T and incubated with streptavidin-peroxidase (Sigma-Aldrich, St. Louis, MO, USA) at 4°C for 1 h. After washing, tetramethylbenzidine (TMB) substrate solution (KPL, Gaithersburg, MD, USA) was added, and the reaction was stopped with TMB stop solution (KPL). Optical density was determined at 450 nm with a Synergy 2 multimode microplate reader (BioTek Instruments, Winooski, VT, USA). The association constant (K_A_, 1/μM SA) values were determined by fitting the data to the “one site-total binding” equation by using nonlinear regression in Prism 6.0 software (GraphPad Software, La Jolla, CA, USA). The reported data represent the mean of at least four individual and independent experiments for each virus.

### Direct and competitive antibody-binding assays

The binding of viruses by MAbs was measured by enzyme-linked immunosorbent assay (ELISA). Briefly, purified and concentrated viruses were applied to Immulon 2HB microtiter plates (Thermo Scientific, Rochester, NY, USA) at 1 μg/well. The virus-coated plates were blocked with 1% BSA in PBS and then incubated with each MAb (1:200 dilution, ~2.5 μg/ml) in 1% BSA in PBS-T. After an additional incubation with peroxidase-conjugated goat anti-mouse IgG (Sigma-Aldrich, St. Louis, MO, USA), the signal was developed using TMB as the substrate. The reaction was stopped with TMB stop solution, and optical density was determined at 450 nm. The values of the selected H1N1 viruses were normalized to those of the wild-type CA/04 strain. Two independent assays were performed, and each assay was run in duplicate wells.

The competitive assay was based on the competition for binding sites on the viral particle between nonlabeled MAb and biotinylated polyvalent synthetic sialoglycoconjugates [18]. Briefly, the virus-coated plates (1 μg/well for each virus) were blocked with 1% BSA in PBS and then incubated with fixed amount of each sialylglycopolymer (~ 10 × 1/K_A_) in the presence of MAb 28668 (1:100 dilution, 5 μg/ml) in 1% BSA in PBS-T at 4°C for 1 h. Plates were then washed with cold PBS-T and incubated with streptavidin-peroxidase at 4°C for 1 h. After washing, TMB substrate solution was added and the reaction was stopped with TMB stop solution after 30 min. Optical density was determined at 450 nm and the values for each virus were normalized to the respective values in the absence of MAb. The reported data represent the mean of at least three individual experiments for each virus.

### NA enzyme activity and kinetics

The NA activity of influenza H1N1 viruses was measured by a fluorescence-based assay using the fluorogenic substrate MUNANA (Sigma-Aldrich, St Louis, MO, USA), based on the method of Potier et al. [19] as described previously [20]. Briefly, H1N1 viruses were standardized to an equivalent NA protein content of 0.015 ng/μl as determined by protein gel electrophoresis using purified and concentrated viruses (S1 Fig). This virus dilution was selected as a dilution that converted ≤15% MUNANA substrate to product during the reaction time in order to meet the requirements for steady-state kinetic analysis [20]. Virus dilutions were prepared in enzyme buffer [32.5 mM of 2-(N-morpholino) ethanesulfonic acid (MES), 4 mM of calcium chloride, pH 6.5] and added (100 μl/well) in duplicate to a flat-bottom 96-well opaque black plate (Corning, Tewksbury, MA, USA). After pre-incubation for 20–30 min at 37°C, the MUNANA substrate at various concentrations (separately pre-incubated for 20–30 min at 37°C) was added to all wells (50 μl/well). Immediately after adding the MUNANA substrate, the plate was transferred to a 37°C pre-warmed SpectraMAX Gemini XPS microplate reader (Molecular Devices, Sunnyvale, CA, USA) and fluorescence was measured every 60 s for 60 min at 37°C, using excitation and emission wavelengths of 360 nm and 460 nm, respectively. Enzymatic reactions were performed under conditions where signal-to-noise ratios were above 10 during more than 30 min of the reaction time. Time course data from each concentration of the MUNANA substrate were examined for linearity by linear regression analysis. Data with *R*^2^ > 0.99 were used for analysis. The kinetic parameters Michaelis-Menten constant (K_m_) and maximum velocity of substrate conversion (V_max_) of the NAs were calculated by fitting the data to the appropriate Michaelis-Menten equation by using nonlinear regression in Prism 6.0 software (GraphPad Software, La Jolla, CA, USA). Values are the means of three independent determinations.

### Lectin binding and viral receptor binding assays using surface plasmon resonance

Biacore T200 biosensor (GE Healthcare, Chicago, IL, USA) was used to evaluate steady-state affinity constants of MAA and SNA lectins. Briefly, polyvalent biotinylated glycoconjugates 6′SLN and 3′SLN (Consortium for Functional Glycomics, 30 kDa) were diluted in HBS-P buffer (0.01 M HEPES pH 7.4, 0.15 M NaCl, 3 mM EDTA, 0.005% v/v Surfactant P20) and immobilized on the steptavidin-coated Biacore chip to a maximum response of approximately 300–400 resonance units (RU). Non-influenza binding polyvalent glycan, GD3, (Neu5Acα2-8Neu5Acα2-3Galβ1-4Glcβ-SpNH-LC-LC-biotin) was immobilized on the reference flow-cell at the same level and served as a buffer mismatch for a non-specific binding control. Lectins serially diluted in HBS-P buffer to a concentration range of 1–150 nM were applied over immobilized glycans for 2 min at a flowrate of 30 μl/min. Regeneration of the surface between each cycle was performed using single 30 sec pulse with 50 mM NaOH. Binding level (RU) was measured for each lectin concentration (μM) and resulted in experimental data that were fitted by non-linear regression analysis using BIAevaluation software 4.1. Steady-state association constants were calculated based on best-fit results using projected maximum binding capacity (R_max_) and 1:1 interaction model. Dissociation constant (K_D_, μM) was calculated as a reciprocal of association constant (K_A_, 1/μM) for each lectin.

Purified and concentrated viruses serially diluted in HBS-P buffer in the presence or absence of NA inhibitor, 5 mM zanamivir, to a concentration range of 0.005 nM-0.16 nM were applied over immobilized 6′SLN or 3′SLN glycans at a flowrate of 30 μl/min for 2 min. α2,8-linked SA receptor, GD3, was used as a negative control for virus binding. Binding level was measured as RU signal 10 sec after the end of the association phase. Dissociation was allowed to proceed for 10 min before regeneration. Regeneration was performed using 30 sec pulse of 50 mM NaOH at 50 μl/min flowrate. Biacore sensograms were normalized by subtraction of the signal in control flowcell and a signal from zanamivir control. Association rate (k_on_) and dissociation rate (k_off_) constants together with equilibrium association (K_A_) and dissociation (K_D_) constants were calculated using BIAevaluation software 4.1.

### Statistical analysis

Plaque size and number in MDCK cells, virus yield in MDCK and Calu-3 cells, binding to SA receptors and MAbs, and NA enzyme kinetic parameters (K_m_ and V_max_) of the wild-type and mutant H1N1 influenza viruses were compared by *t*-test or by analysis of variance (ANOVA). Probability values ≤ 0.05 indicate statistically significant differences.

## Results

### *In vitro* selection of H1N1 mutants in Calu-3 cells

**Fig 1:**
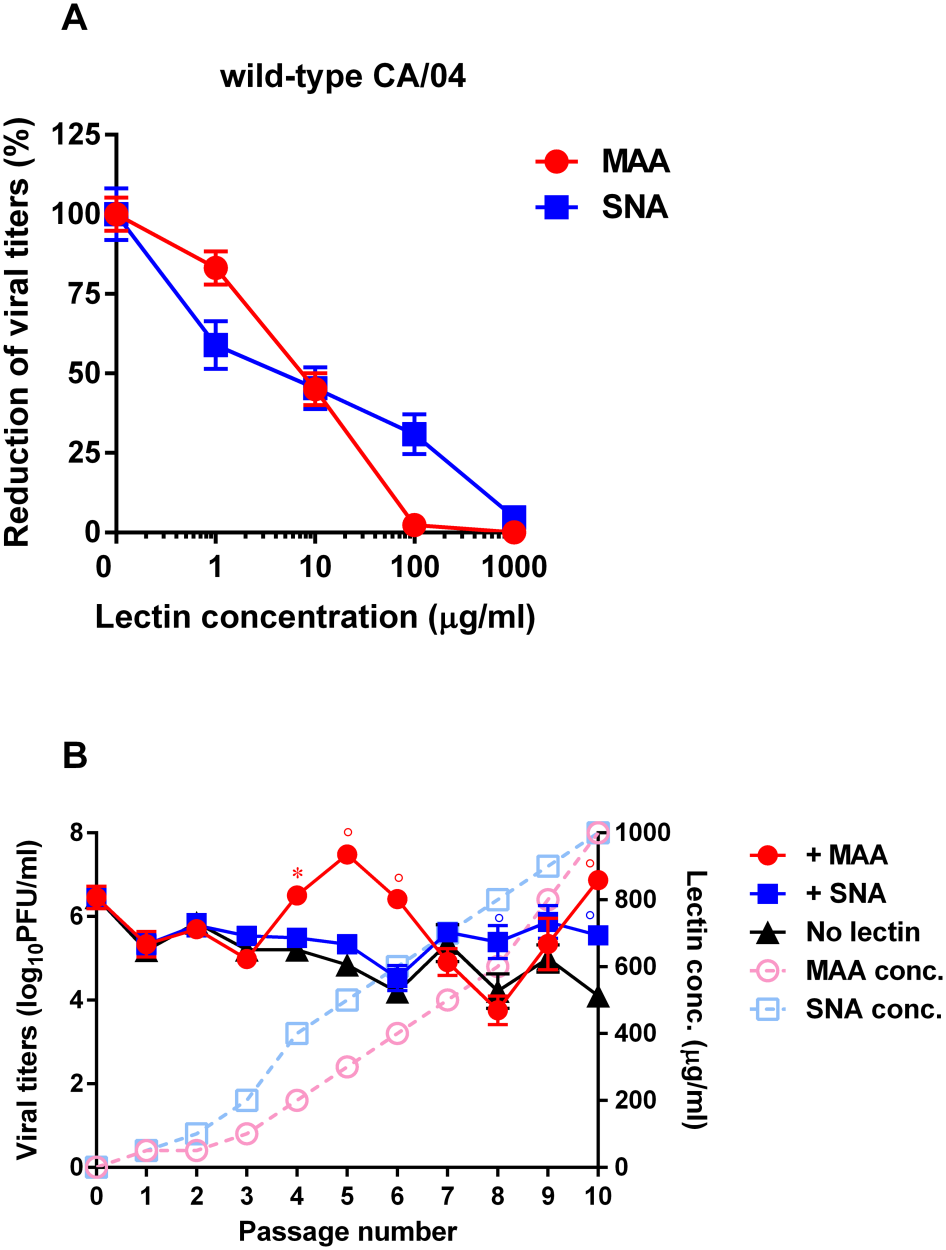
**(A) Antiviral activity of MAA and SNA lectins against the wild-type CA/04 virus as determined by virus reduction assay in Calu-3 cells. (B) Generation of influenza A viruses with decreased sensitivity to MAA and SNA lectins.** CA/04 virus was passaged in Calu-3 cells in the presence or absence of increasing concentrations of MAA or SNA lectins. *P < 0.05, °*P* < 0.01, compared to the values for the CA/04 virus.

We first measured the sensitivity of influenza CA/04 virus to pretreatment with MAA and SNA lectins. We evaluated the activity of the lectins by virus reduction assay using Calu-3 cells as target cells. CA/04 virus was sensitive to pretreatment with MAA or SNA: mean EC_50_ ≈ 7.9 ± 1.3 μg/ml (Fig 1A). We then serially passaged the CA/04 strain 10 times in Calu-3 cells in the presence of increasing concentrations of MAA and SNA to provide an opportunity for selection of viral receptor variants (Fig 1B). Virus yields were measured by plaque assay using MDCK cells after each passage. To monitor the emergence of any adaptive amino acid changes due to repeated passage in Calu-3 cells, we also passaged the parental virus in parallel without any selective pressure. Analysis of the viral titers of the viruses cultured in the presence of increasing concentrations of lectins showed the largest increase in plaque number at passage 10 compared to that of the virus passaged in the absence of lectins (≈ 2.1 ± 0.9 log_10_PFU/ml, Fig 1B).

### Lectin sensitivity and growth characteristics of selected H1N1 variants

**Fig 2:**
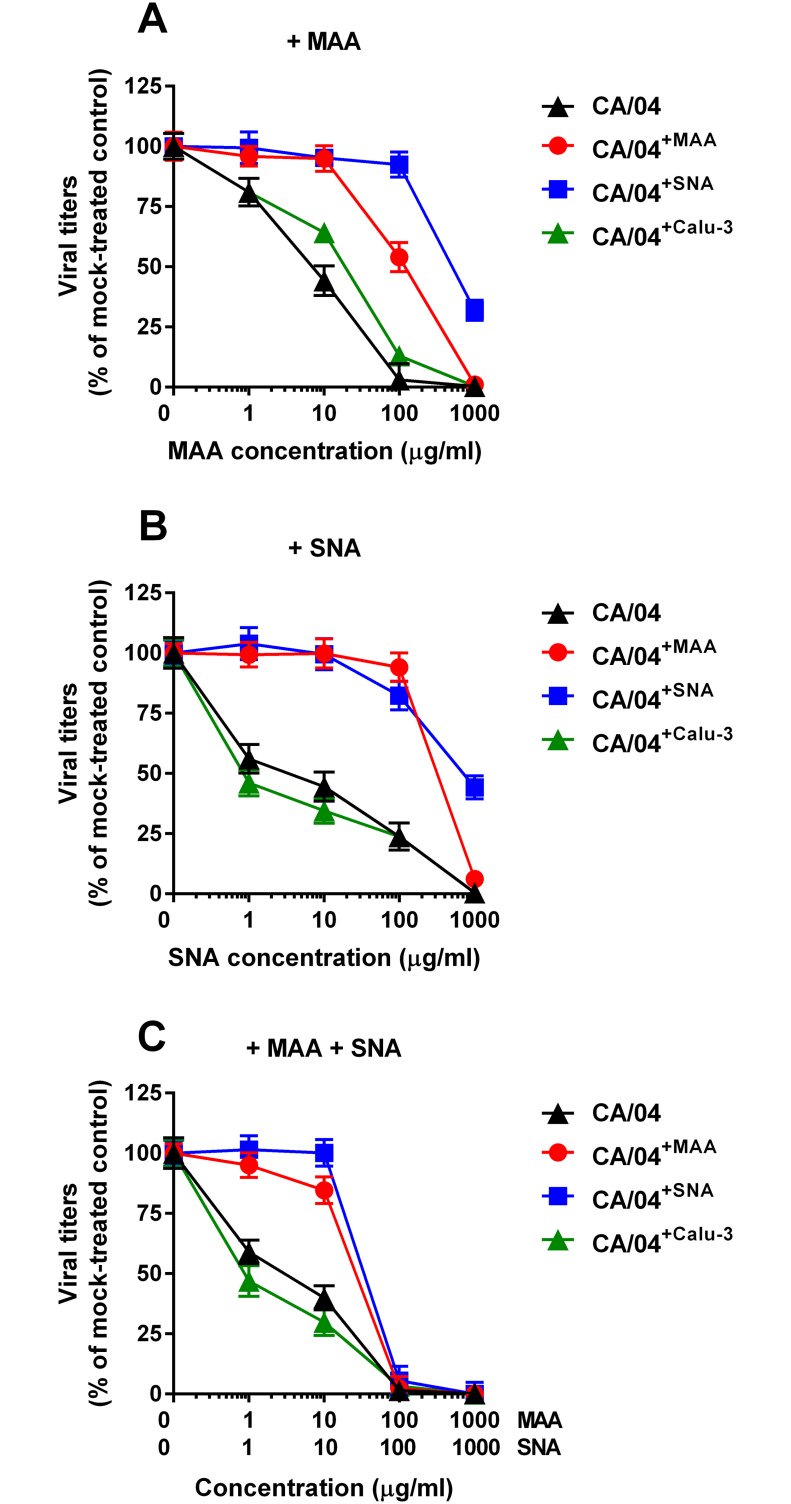
Antiviral activity of MAA (A), SNA (B), and combination of both lectins (C) against the CA/04, CA/04^+MAA^, CA/04^+SNA^, and CA/04^+Calu-3^ viruses as determined by virus reduction assay in Calu-3 cells.

After 10 serial passages in the presence or absence of lectins, we prepared stocks of three selected H1N1 variants: CA/04^+MAA^, CA/04^+SNA^, and CA/04^+Calu-3^ by plaque purification in MDCK cells. We then tested their sensitivity to both lectins and their combination by virus yield reduction assay in Calu-3 cells. As shown in Fig 2A and 2B, sensitivity of the CA/04^+Calu-3^ variant to both lectins differed slightly (≈ 5-fold) from that of the wild-type virus. In contrast, sensitivities of CA/04^+MAA^ and CA/04^+SNA^ variants to MAA and SNA differed significantly from that of CA/04^+Calu-3^ and the wild-type viruses. Surprisingly, the CA/04^+MAA^ variant exhibited markedly reduced sensitivity not only to MAA (EC_50_ = 108.8 ± 12.0 μg/ml), but also to SNA (EC_50_ = 551.4 ± 60.7 μg/ml). CA/04^+SNA^ also demonstrated diminished sensitivity to both lectins compared to CA/04 (EC_50_ ≈ 796.8 ± 94.0 μg/ml). These findings indicated that both CA/04^+MAA^ and CA/04^+SNA^ viruses may have acquired one or more mutation(s) that decreased their sensitivity to the lectin pretreatment. We also observed that pretreatment with the combination of both lectins led to a stronger reduction of virus yields of CA/04^+MAA^ and CA/04^+SNA^ as compared to either lectin alone (Fig 2C).

**Fig 3:**
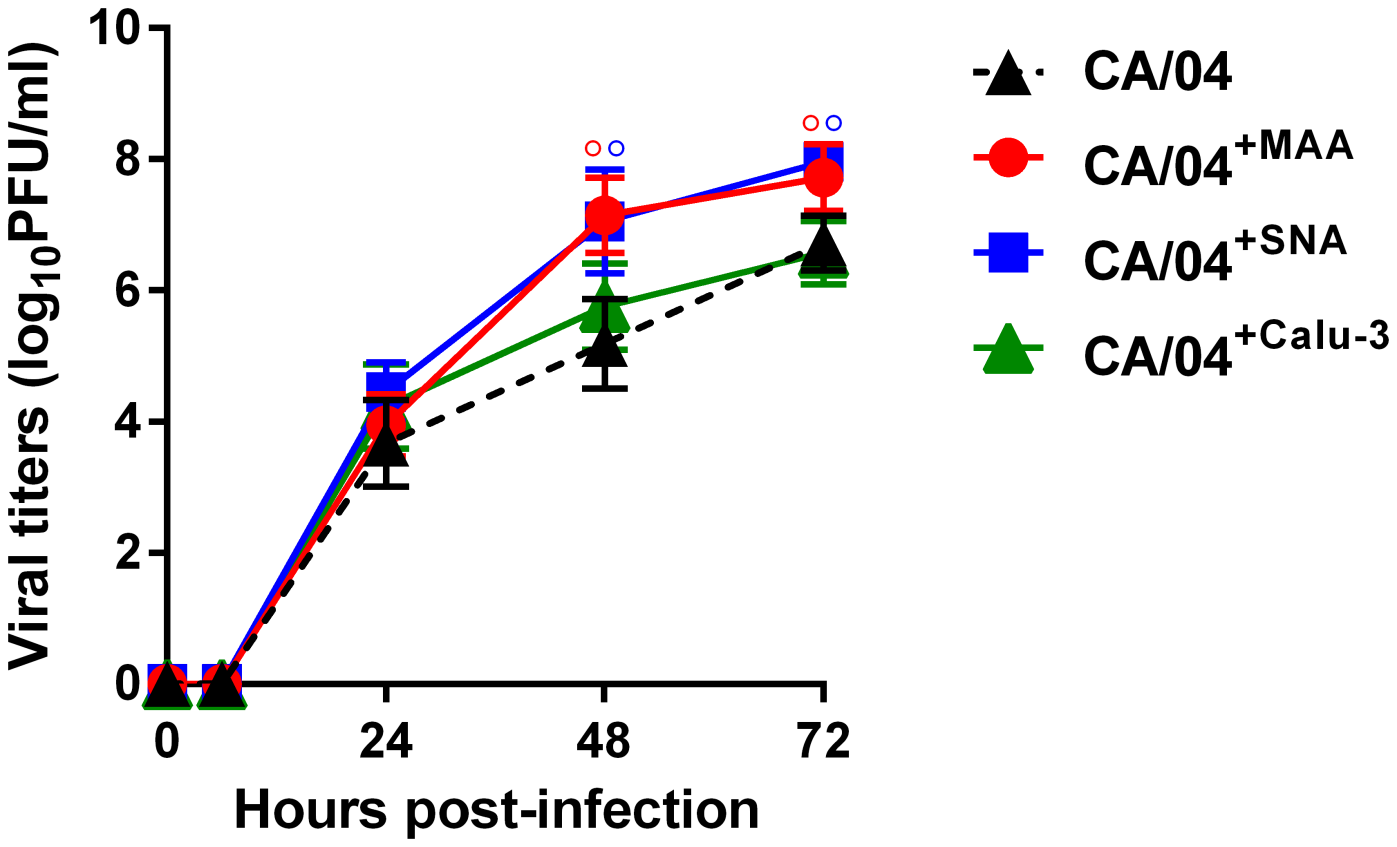
Replication of the CA/04, CA/04^+MAA^, CA/04^+SNA^, and CA/04^+Calu-3^ viruses in Calu-3 cells. The results are expressed as log_10_PFU/ml from three to four independent experiments performed on different days. °*P* < 0.01, compared to the values for the wild-type CA/04 virus.

We next examined growth of the wild-type, CA/04^+MAA^, CA/04^+SNA^, and CA/04^+Calu-3^ viruses in MDCK cells. CA/04^+MAA^ variant grew to significantly higher titers than the parental strain, and CA/04^+MAA^ and CA/04^+SNA^ formed larger plaques than CA/04 (*P* < 0.05, data not shown). To further evaluate the replicative ability of the selected H1N1 variants, we assayed their virus yields in comparison to those of the parental strain after multiple replication cycles in Calu-3 cells. As shown in Fig 3, the CA/04^+MAA^ and CA/04^+SNA^ viruses grew to significantly higher titers than the wild-type virus at 48 and 72 h post-infection (*P* < 0.01). This finding indicates that sequential passage of the parental H1N1 virus in Calu-3 cells in the presence of lectins promoted selection of variants with enhanced growth properties.

### Sequence analysis of selected H1N1 variants

To identify amino acid changes acquired by the CA/04 virus after culture in the presence or absence of lectins, we sequenced the complete genomes of our selected variants at the end of the passaging protocol (Table 1). Sequence analysis mapped the acquired mutations to the HA1 and NA proteins. A total of 4 amino acid substitutions and 1 nucleotide change were identified. We found that passage of CA/04 in the presence of MAA resulted in development of two mutations in HA1 (G155E and S183P, H1 numbering used throughout the text) and one mutation in NA (G201E, N1 numbering used throughout the text). Sequence analysis of the genome of the CA/04^+SNA^ virus revealed three mutations in HA1 (N129D, G155E, and S183P). The S183P HA1 mutation was selected when CA/04 was passaged in the absence of lectins (Table 1). To determine if the amino acid changes identified in our CA/04^+MAA^, CA/04^+SNA^, and CA/04^+Calu-3^ variants might also be present in other pandemic 2009 H1N1 isolates, we analyzed ~26,695 H1N1 genomic sequences deposited in the Influenza Research Database (data were obtained from the National Institute of Allergy and Infectious Diseases (NIAID) database: www.fludb.org). The N129D or G155E HA1 amino acid substitutions were relatively infrequent and they were present in ~0.4% of the related H1N1 strains in the Influenza Research Database. In contrast, the number of human isolates expressing the S183P mutation was ~ 5.4% between 2009 and 2011, and rapidly increased in 2017 up to ~36.4% (S2 Fig). The G201E NA mutation was found in ~0.1% of all H1N1 viruses examined.

**Table 1:** Nucleotide and amino acid substitutions identified in selected H1N1 influenza viruses.

Proteins	CA/04^+MAA^	CA/04^+SNA^	CA/04^+Calu-3^
HA^a^	G155E, S183P	N129D, G155E, S183P	S183P
NA^a^	G201E, *A1026G*	–	–

^*a*^ H1 and N1 numbering.

Italic indicates nucleotide change.

### Effects of HA1 amino acid substitutions on receptor specificity

**Fig 4:**
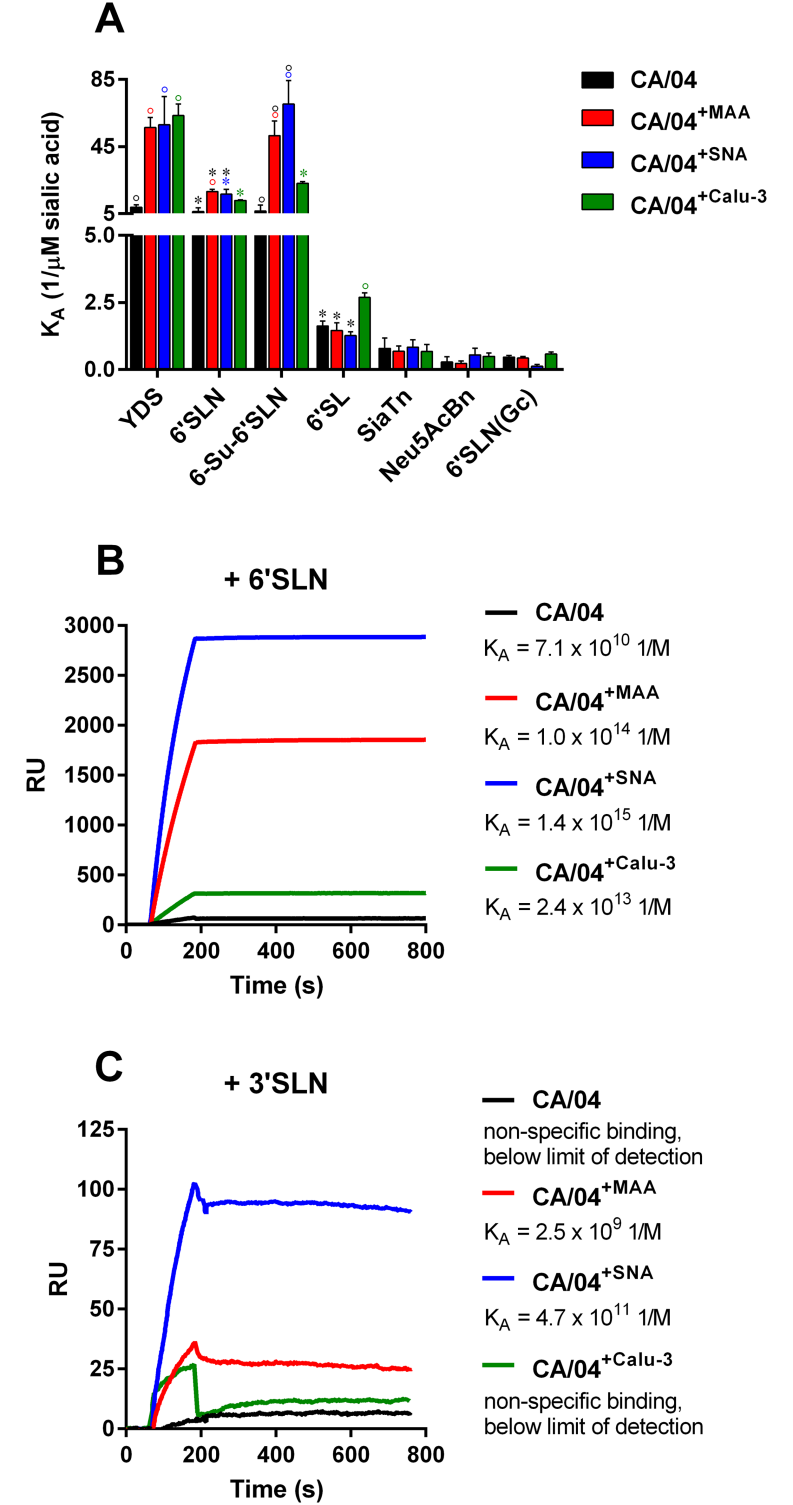
**(A) Receptor specificity of the CA/04, CA/04**^**+MAA**^**, CA/04**^**+SNA**^**, and CA/04**^**+Calu-3**^ **viruses.** °*P* < 0.01, *P < 0.05 in color, compared to the values for the wild-type CA/04 virus. °*P* < 0.01, *P < 0.05 in black, compared to the values for the CA/04^+Calu-3^ virus. **Biacore sensograms for CA/04, CA/04**^**+MAA**^**, CA/04**^**+SNA**^**, and CA/04**^**+Calu-3**^ **binding with 6'SLN- (B) or 3'SLN-immobilized sensor surface (C).** Purified and concentrated viruses serially diluted in HBS-P buffer in the presence of 5 mM zanamivir to a concentration range of 0.005–0.16 nM were applied over immobilized glycans. Binding level (RU) at the highest virus concentration (0.16 nM) is shown. Association rate (k_on_) and dissociation rate (k_off_) constants together with equilibrium association (K_A_) and dissociation (K_D_) constants (S2 Table) were calculated using BIAevaluation software 4.1.

To determine if the HA1 mutations that we identified affect the affinity of HA for sialic receptors, we examined the receptor specificity of three selected variants, CA/04^+MAA^, CA/04^+SNA^, and CA/04^+Calu-3^, in comparison to the wild-type virus (Fig 4A). The chemical structures of the sialic acid receptors are shown in S1 Table. All three variants showed significantly higher binding to the extended version of the “human-type” receptor, YDS (↑~6.8-fold, *P* < 0.05). The CA/04^+Calu-3^ variant demonstrated higher affinity for 6′SL receptor compared to the rest of the viruses (↑1.9-fold, *P* < 0.05). Based on the K_A_ values, both the CA/04^+MAA^ and CA/04^+SNA^ variants exhibited higher affinity for the major analog of the human receptor, 6′SLN, and its sulfated version, 6-Su-6′SLN, compared to CA/04 and CA/04^+Calu-3^ (↑4.1-fold, *P* < 0.05).

Since the binding level to the “avian-type” receptor, 3′SLN, was at the detection limit of our assay among all tested viruses (data not shown) [12], we studied the binding pattern of the H1N1 variants to 3′SLN glycan in comparison with 6′SLN in the presence of NA inhibitor, zanamivir, using Biacore surface plasmon resonance biosensor (Fig 4B and 4C). Our data showed that both the CA/04^+MAA^ and CA/04^+SNA^ variants had higher association rate constants of binding with 6′SLN as compared to CA/04 and CA/04^+Calu-3^ (k_on_ ≈ 7.5 x 10^7^ M^-1^ x s^-1^; S2 Table). However, since CA/04^+SNA^ had a 15-fold lower dissociation rate constant (k_off_) compared to CA/04^+MAA^, the CA/04^+SNA^ virus showed higher equilibrium association constant K_A_ with 6′SLN (Fig 4B, S2 Table). Importantly, we also observed that, whereas binding of CA/04 and CA/04^+Calu-3^ with 3′SLN was negligible, both CA/04^+MAA^ and CA/04^+SNA^ viruses were able to bind the “avian-type” receptor. Moreover, CA/04^+SNA^ exhibited ~188-fold higher affinity (K_A_) for 3′SLN compared to CA/04^+MAA^ (Fig 4C, S2 Table).

### Effects of HA1 amino acid substitutions on antigenicity and competition between MAb 28668 and sialic receptors

Having identified HA1 mutations by *in vitro* selection that contributed to a change in receptor specificity, we assessed their potential impact on antigenicity. We used goat and ferret antisera and MAbs raised against a selection of human, pandemic, and swine H1 isolates to determine the antigenic properties of the CA/04, CA/04^+MAA^, CA/04^+SNA^, and CA/04^+Calu-3^ viruses (Table 2). None of the viruses reacted with the MAbs raised against 1918 pandemic or swine H1 viruses (data not shown). The results of the HI assay showed that both CA/04^+MAA^ and CA/04^+SNA^ variants had a reduced ability to react with goat and ferret antisera and two MAbs, 28665 and 28668, raised against the parental and closely related H1N1 strains (8- to 256-fold differences compared to CA/04). CA/04^+MAA^ and CA/04^+SNA^ were not neutralized by these MAbs in MN assay in MDCK cells (Table 2).

**Table 2:** Antigenic characterization of wild-type and selected H1N1 influenza viruses by hemagglutination inhibition and microneutralization assays.

H1N1 virus	Titer							
	Goat antiserum against CA/04	Ferret antiserum against:			MAbs against CA/04:			
		CA/04	A/CA/07/09	A/TN/1-560/09	28665	28666	28667	28668
CA/04	1280^a^	1280	1280	1280	6400^a^/6400^b^	51200/>204800	800/6400	12800/102400
CA/04^+MAA^	160	10	80	10	<200/<200	51200/>204800	400/6400	<50/<200
CA/04^+SNA^	160	10	160	10	<200/<200	51200/>204800	400/3200	50/200
CA/04^+Calu-3^	1280	1280	1280	1280	1600/6400	51200/>204800	400/3200	12800/51200

^a^ Values are expressed as reciprocals of serum/antibody dilutions that inhibited 8 hemagglutination units of H1N1 influenza virus.

^b^ Neutralizing antibody titers (arithmetic mean) to H1N1 viruses were titrated in MDCK cells.

**Fig 5:**
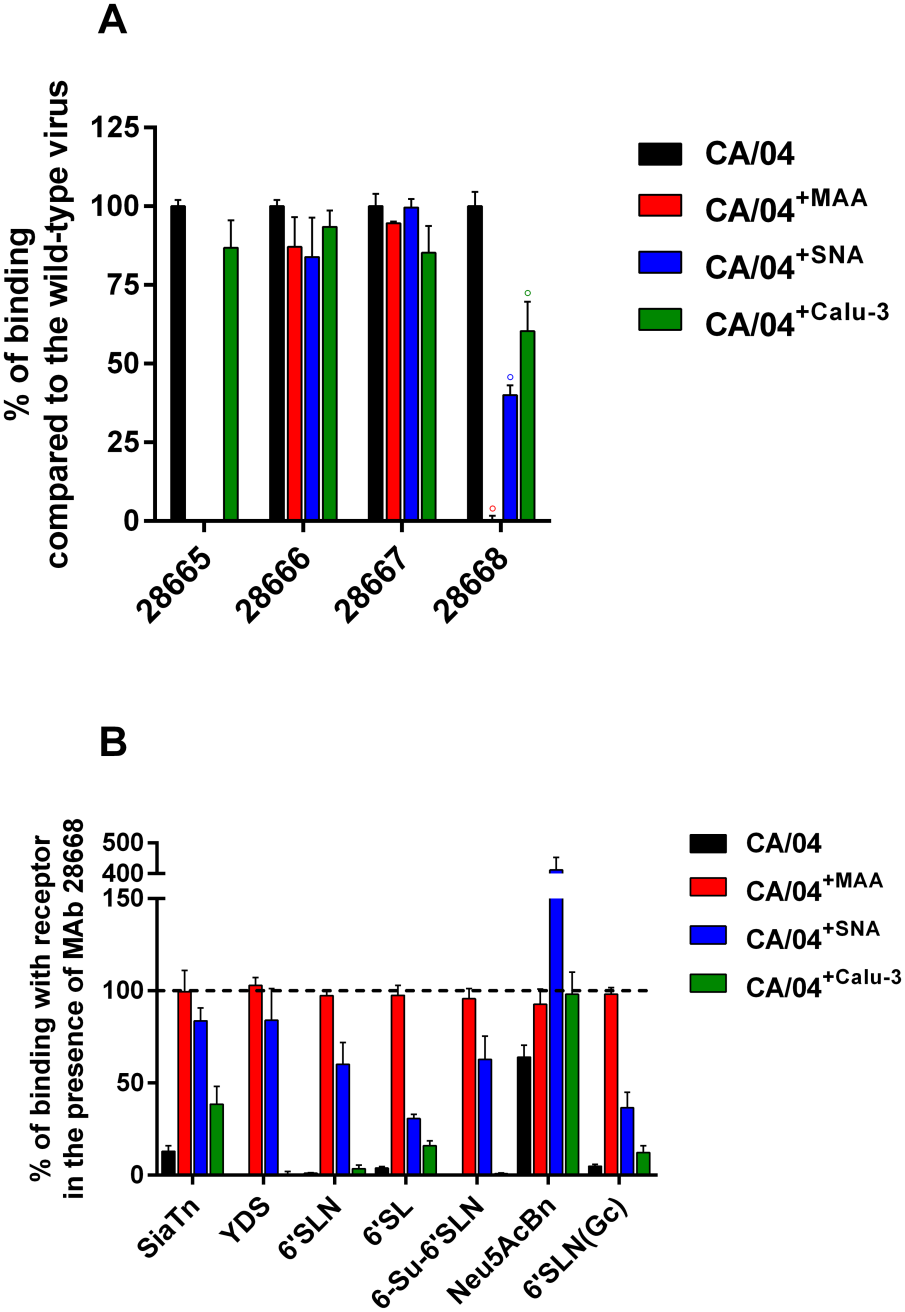
**(A) Binding of CA/04, CA/04**^**+MAA**^**, CA/04**^**+SNA**^**, and CA/04**^**+Calu-3**^ **by a panel of MAbs specific for HA1 of the wild-type CA/04 virus.** Optical density values generated with mutant viruses were normalized to those of CA/04**. (B) Receptor-binding activity of CA/04, CA/04**^**+MAA**^**, CA/04**^**+SNA**^**, and CA/04**^**+Calu-3**^ **in competitive reaction with biotinylated polyvalent synthetic sialoglycoconjugates and the MAb 28668.** Optical density values for each virus were normalized to the respective values in the absence of the MAb.

We next investigated whether differences in HI and MN titers between the wild-type and CA/04^+MAA^ and CA/04^+SNA^ variants were due to a direct effect of the MAb binding. We measured the binding of the MAbs raised against the wild-type CA/04 virus to CA/04, CA/04^+MAA^, CA/04^+SNA^, and CA/04^+Calu-3^ by ELISA. Two MAbs, 28666 and 28667, reacted strongly with all four viruses (Fig 5A). The MAb 28665 demonstrated significant loss of binding to CA/04^+MAA^ and CA/04^+SNA^, which was in a good correlation with the HI data (Table 2). However, despite the fact that the MAb 28668 neither inhibited hemagglutination nor neutralized CA/04^+MAA^ and CA/04^+SNA^, this MAb did not recognize CA/04^+MAA^, but was able to bind the CA/04^+SNA^ variant in the ELISA assay.

We next performed competitive binding assays between the non-labeled MAb 28668 and biotinylated polyvalent synthetic sialoglycoconjugates to determine if they were able to compete for HA binding sites on the viral particle (Fig 5B). Since the MAb 28668 did not bind to CA/04^+MAA^ virus (Fig 5A), it was not able to compete with any of the tested sialic receptors, and as a consequence, CA/04^+MAA^ exhibited similar binding to all receptors with or without the MAb. In contrast, since the MAb 28668 possessed high affinity for the wild-type CA/04 virus and CA/04^+Calu-3^, it efficiently displaced the majority of the receptor molecules from the viral binding sites, except Neu5AcBn receptor. However, despite the fact that the MAb 28668 bound to the CA/04^+SNA^ variant, it was unable to displace any of the receptor from the CA/04^+SNA^ HA binding sites, and moreover, it unexpectedly increased binding to the Neu5AcBn polymer (Fig 5B).

### Effects of NA amino acid substitutions on NA activity and specificity

**Fig 6:**
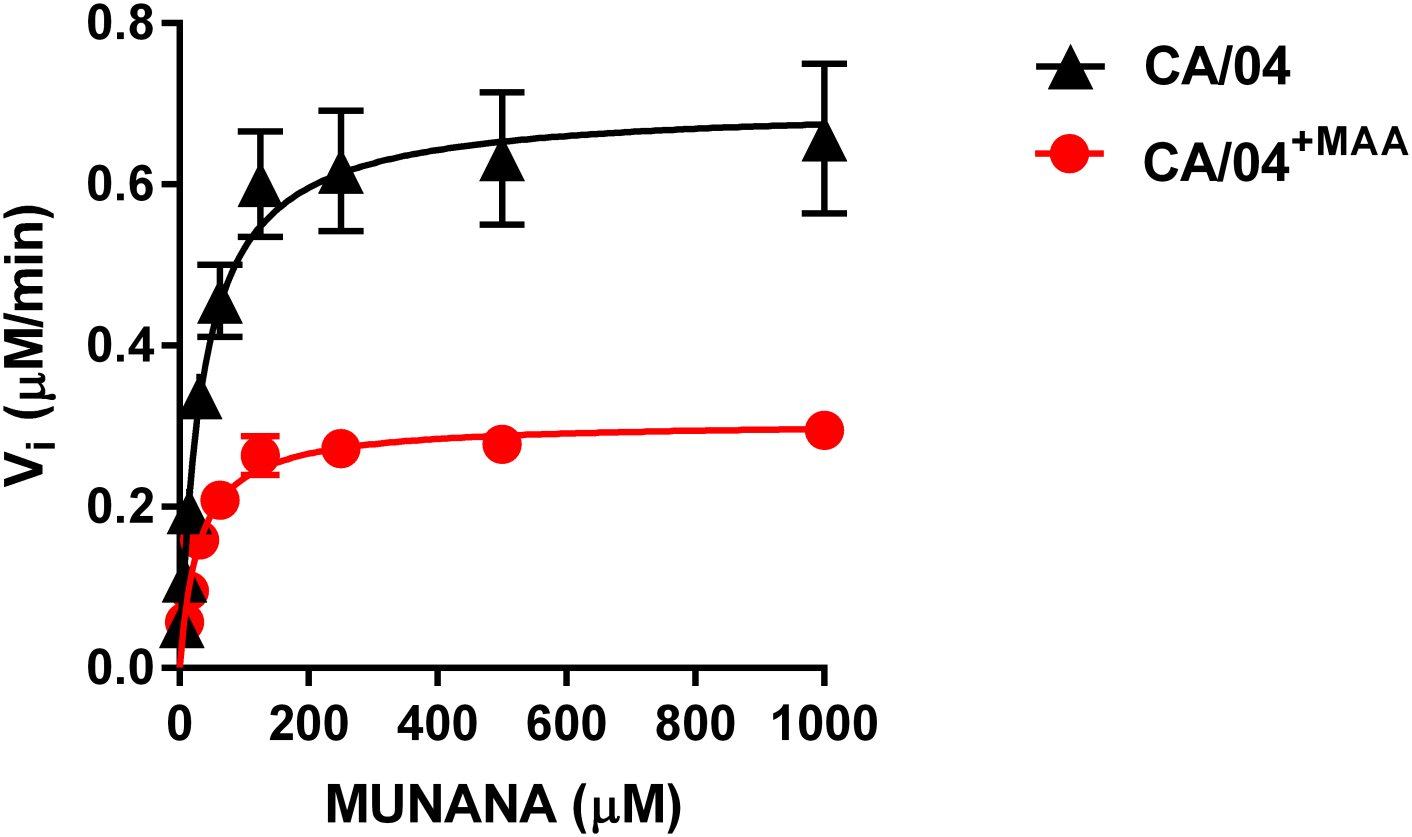
NA enzyme kinetics of the CA/04 and CA/04^+MAA^ viruses. Substrate conversion velocity (V_i_) of NA was measured as a function of substrate concentration.

To evaluate the impact of the G201E mutation identified in the CA/04^+MAA^ variant on NA enzyme activity, we determined the NA enzyme K_m_ and V_max_ values for the CA/04 and CA/04^+MAA^ viruses using fluorogenic MUNANA as a substrate. We observed that although the G201E mutation had no effect on the K_m_ value, it decreased NA enzyme activity (V_max_ ratio relative to the wild-type virus = 0.4; Fig 6 and Table 3). We next used Biacore surface plasmon resonance to study the binding of the H1N1 viruses to 6′SLN and 3′SLN in the presence of uninhibited NA (S3 Fig and S3 Table). Our results showed a decrease in association rate constants (k_on_), a concomitant increase in dissociation rate constants (k_off_), and, as a consequence, a significant decrease in equilibrium association constants (K_A_) compared to the respective values measured when NA protein was inhibited (S2 Table). The only H1N1 variant which demonstrated almost similar k_on_, k_off_, and K_A_ values determined with and without zanamivir was the CA/04^+MAA^ virus bound to 3′SLN glycan (S2 and S3 Tables). Thus, our data indicate that the N1 NA carrying mutation, G201E, reduced NA ability to cleave 3′SLN glycan immobilized on the sensor surface, clearly suggesting that the catalysis of 3′SLN was diminished by the mutant N1 protein.

**Table 3:** Enzymatic properties of the neuraminidase of recombinant H1N1 influenza viruses.

Viruses	V_max_ (μM/min)^a^	K_m_ (μM)^b^
CA/04	0.70 ± 0.02	34.35 ± 3.77
CA/04^+MAA^	0.31 ± 0.01	29.89 ± 2.74

^*a*^ The V_max_ was calculated using a nonlinear regression of the curve according to the Michaelis-Menten equation.

^*b*^ The K_m_ represents the Michaelis-Menten constant (μM) at which the reaction rate is half of V_max_. The enzyme kinetic data were fit to the Michaelis-Menten equation using GraphPad Prism, version 6.0. Values are the means ± standard deviations from three independent determinations.

### Carbohydrate-binding specificities of the MAA and SNA lectins

**Fig 7:**
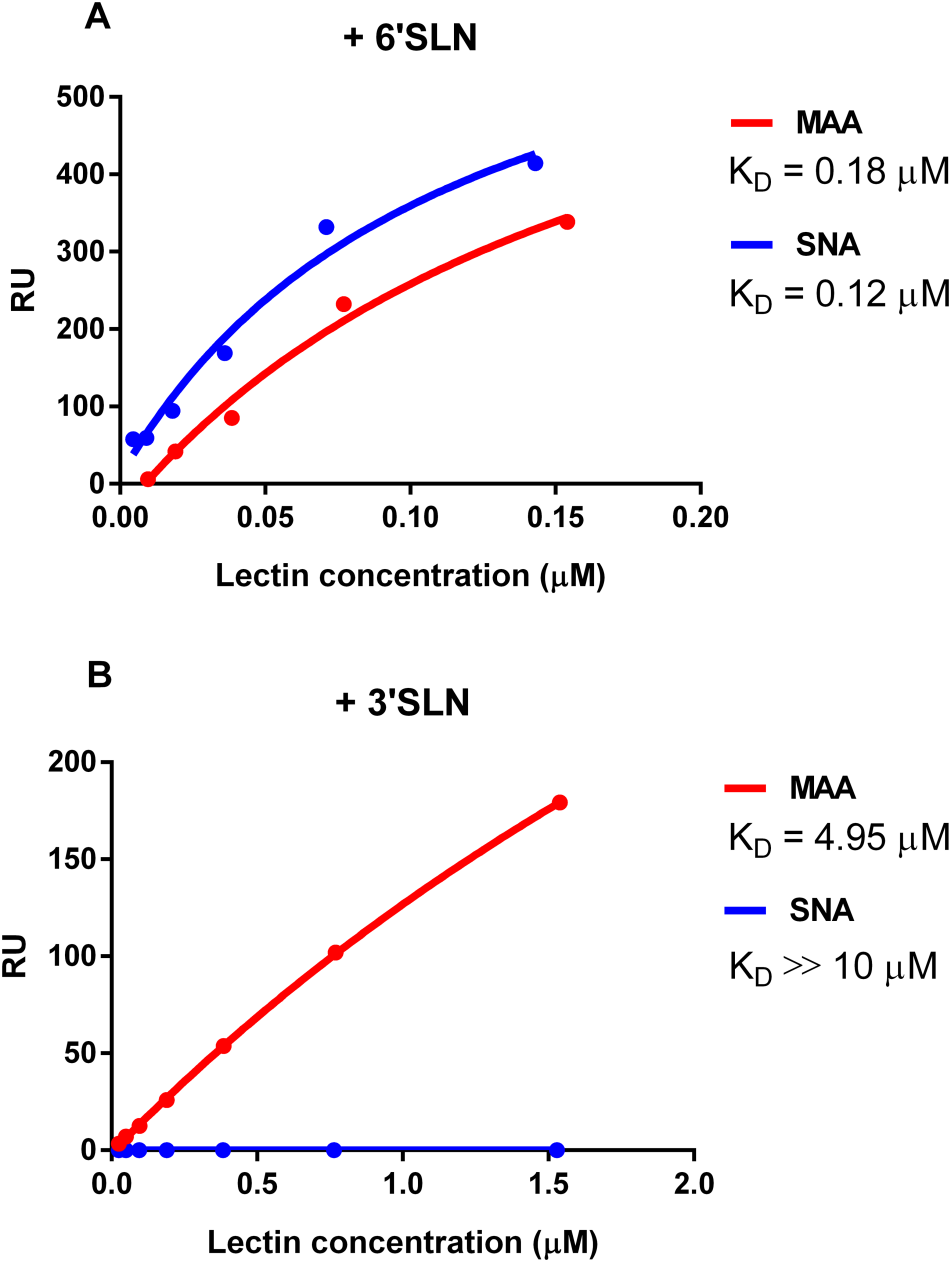
MAA and SNA binding towards 6′SLN (A) and 3′SLN (B) glycans as determined by Biacore surface plasmon resonance.

Previous studies demonstrated that the technical specifications provided by commercial vendors sometimes cite lectin binding specificities that differ from those defined in direct scientific studies [21–23]. Since our results showed that viruses cultured in the presence of either lectin exhibited markedly reduced sensitivity to both lectins, which may be explained by actual lectin binding properties, we examined the carbohydrate specificities of both the MAA and SNA lectins used in this study by Biacore surface plasmon resonance (Fig 7). We found that, in contrast to the vendor’s specification for α2,3-linked SAs, MAA bound both 3′SLN and 6′SLN glycans (K_D_ = 0.18 μM for 6′SLN; K_D_ = 4.95 μM for 3′SLN). This finding is in agreement with the previous study by Nicholls et al. [23], which showed that EY Laboratories, Inc. provides an undefined mixture of *Maackia amurensis* agglutinin MAA containing unequal amounts of MAA-1 and MAA-2. MAA-1 binds to terminal Siaα2-3Galβ1-4GlcNAc moieties on N-linked glycans, and MAA-2 has specificity for O-linked glycans containing the trisaccharide Siaα2-3Galβ1-3GalNAc [21,23]. Since Calu-3 cells were reported to have a high abundance of O-linked α2,3 SAs [24], these sialic receptors should be masked after MAA treatment containing the MAA-2 isoform. Furthermore, our data corroborated with the data posted on the public website of the Consortium for Functional Glycomics (http://www.functionalglycomics.org/), which demonstrated that MAA from EY Laboratories, Inc. can also bind α2,6-linked glycans. Using Biacore surface plasmon resonance, we observed that SNA lectin exhibited single α2,6-linked SA carbohydrate-binding specificity (K_D_ = 0.12 μM; Fig 7A).

## Discussion

The 2009 pandemic H1N1 strain, CA/04, was isolated from a pediatric patient with uncomplicated upper respiratory tract illness, and infections with this virus have been mostly mild [11]. Mortality was usually associated with high risk groups, including those with chronic lung and heart conditions, obesity, and pregnancy [25,26]. However, some deaths have occurred in individuals with no known underlying conditions, and recovery of some critically ill children has resulted in neurological sequelae [26,27]. Recent studies demonstrated that HA receptor binding to α2,3-linked SAs, which is predominantly present in the human lower respiratory tract, is necessary for increased virulence in mice and possibly in humans [28,29]. Therefore, extensive circulation of 2009 pandemic H1N1 viruses raises the concern of a potential shift toward α2,3-linked “avian-like” receptor specificity. In this study, we applied an *in vitro* selection approach using two plant lectins, MAA and SNA, that bind to glycolipids and glycoproteins containing SA α2,3 or α2,6 configurations, to understand the potential for genetic changes of the 2009 pandemic H1N1 virus to increase HA receptor binding toward α2,6- or α2,3-linked glycans.

Our *in vitro* selection study led to the identification of three amino acid substitutions in the HA protein (N129D, G155E, and S183P) that affected the H1 HA receptor binding specificity. The N129D mutation is located outside the major antigenic site, Sa, where the amino acid 155 was previously mapped to. The S183P mutation is located within the receptor-binding site, and overlaps with antigenic site, Sb [29,30]. Due to the lack of suitable recombinant H1N1 viruses carrying the single HA1 mutations identified here, it is not yet known which amino acid substitutions or combinations of substitutions are necessary for altering the CA/04 receptor specificity properties. However, having three mutant viruses, CA/04^+MAA^, CA/04^+SNA^, and CA/04^+Calu-3^ that differ from each other only by the presence of one HA1 substitution, made it possible to examine the role of three single amino acid changes. The S183P mutation significantly increased binding to a number of α2,6 SA-linked receptors, including YDS, 6′SL(N), and 6-Su-6′SLN. However, CA/04^+Calu-3^ carrying a single S183P mutation did not show higher replication titers in Calu-3 cells compared to the wild-type CA/04. These results correlate with the previously published findings by O’Donnell et al. [29], and suggest that enhanced “human-like” receptor specificity provides only a minor difference in viral fitness *in vitro* between CA/04 and the mutant CA/04^+Calu-3^ virus. Two other changes, N129D and G155E, were sufficient to significantly increase binding to α2,6-linked glycans, 6′SLN and 6-Su-6′SLN, compared to S183P. Notably, we were able to demonstrate that these HA1 mutations can also increase binding affinity for “avian-like” 3′SLN glycan compared to the wild-type and CA/04^+Calu-3^ viruses using highly sensitive Biacore method. Our findings showed that blocking of the cellular glycans by MAA and SNA lectins with dual and single carbohydrate specificities, respectively, results in the selection of the H1N1 variants with enhanced receptor affinity for both “human-type” and “avian-type” receptors.

Given that positions 129, 155, and 183 are located within or in close proximity to the receptor-binding pocket and major H1 HA antigenic sites, it was anticipated that these mutations would not only modulate receptor binding, but also antigenicity. Our binding analysis demonstrated that, despite the absence of antibody selection, two out of three mutations modified HA antigenicity. The G155E change completely abrogated binding by MAbs 28665 and 28668, and the N129D mutation diminished binding by MAb 28665, but increased affinity to MAb 28668 relative to G155E. Our studies identified N129D and G155E as antigenic mutations. Our findings are consistent with the study by O’Donnell et al. [29], and confirmed that the S183P was an absorptive mutation. The results of our competitive ELISA between MAb 28668 and biotinylated glycans showed that MAb 28668 is incapable of competing with all of the sialyloligosaccharides that we tested. Despite being neutralized by MAb 28668, CA/04 and CA/04^+Calu-3^ were able to bind Neu5AcBn glycan in the presence of this antibody. In contrast, CA/04^+SNA^ bound all sialic receptors in the presence of MAb 28668, and moreover, this virus was able to bind Neu5AcBn to a greater extent in the presence of this antibody than in the absence of this antibody. The loss of competition between the antibody and glycans resulted in a loss of virus neutralization by MAb 28668. Thus, the N129D mutation present only in the CA/04^+SNA^ virus may provide an advantage in overcoming the neutralizing effects of antibody by allowing and/or increasing recognition of host cell receptors even in the presence of such antibodies.

Amino acid changes of the same residues (155 and 183) occurred in the pandemic CA/04 strain in four independent studies either by selection in human airway epithelial cells, mice, or by human antibody [12,28–30]. This indicates that the emergence of these two HA1 mutations is not due to random fluctuation, but rather reflects their positive selection. The 183P residue is common among classical swine and human seasonal influenza viruses isolated from 1998 to 2007, and it was also conserved in the 1918 pandemic H1N1 virus [29]. According to previous studies, the S183P mutation enhances virulence by altering binding to SA receptors in a mouse animal model [28–30]. While it is unknown whether 183P has an effect on virulence in humans, the high frequency of the S183P mutation in contemporary H1N1 viruses in 2017 suggests that this mutation is being strongly selected for in humans.

Our findings indirectly support the hypothesis proposed by Hensley et al. [31] that antigenic drift can be a product of Darwinian selection of mutations throughout the HA1 globular domain that optimize host cell receptor binding avidity, many of which simultaneously alter antigenicity. Our data suggest that influenza virus favors HA1 substitutions that simultaneously change cellular receptor binding and antigenic reactivity. Such HA1 changes are dictated at least in part by HA1 charge and other physical and chemical properties of the glycoprotein. Additionally, our data indicate that HA1 changes with pleiotropic effects involved in antigenic drift can affect functional match between two influenza surface glycoproteins [2,9,10]. Indeed, we observed that the CA/04^+MAA^ virus acquired binding affinity toward “avian-like” 3′SLN glycan; however, the biological significance of the increase in 3′SLN binding remains to be determined. On the other hand, CA/04^+MAA^ acquired the G201E NA mutation, which decreased NA activity in catalyzing 3′SLN and led to efficient virus attachment to this particular sialic receptor. Thus, the G201E NA change compensated for the G155E HA1 mutation and provided an advantage for CA/04^+MAA^ virus growth due to functional compatibility between HA and NA proteins. Our results demonstrate the association between HA1 antigenic changes involved in receptor binding and their modulation of the functional balance between HA and NA.

In conclusion, we have shown that the pandemic CA/04 (H1N1) virus can acquire enhanced receptor affinity for both “human-type” and “avian-type” SA receptors under lectin selective pressure. Such changes in binding affinity are conferred by selection of beneficial HA1 mutations affecting receptor specificity, antigenicity, and/or functional compatibility with the NA protein. It is worth noting, that since one of the new therapeutic strategies to control influenza is masking SA receptors [32,33] from the epithelial surface, such approach might result in enhanced viral receptor specificity to the masked receptors. We consider that the tracking of such changes on the HA1 molecule is important for prediction of possible evolutionary changes of the pandemic H1N1 viruses toward a potentially more virulent form.

## Supporting information

S1 Fig SDS-PAGE gel images of CA/04, CA/04^+MAA^, CA/04^+SNA^, and CA/04^+Calu-3^ viruses (2.5 μg/lane). HA and NA concentrations (%) were determined by optical densitometry and total protein content was determined by BCA protein assay.
(PPTX)

S2 Fig Emergence of the HA N129D, HA G155E, HA S183P, and NA G201E mutations among human pandemic H1N1 influenza viruses between 2009 and 2017. (TIF)

S3 Fig Biacore sensograms for CA/04, CA/04^+MAA^, CA/04^+SNA^, and CA/04^+Calu-3^ binding with 6'SLN- (A) or 3'SLN-immobilized sensor surface (B). Purified and concentrated viruses serially diluted in HBS-P buffer to a concentration range of 0.005–0.16 nM were applied over immobilized glycans. Binding level (RU) at the virus concentration of 0.08 nM is shown. Association rate (k_on_) and dissociation rate (k_off_) constants together with equilibrium association (K_A_) and dissociation (K_D_) constants (S3 Table) were calculated using BIAevaluation software 4.1.
(TIF)

S1 Table Biotinylated sialylglycopolymers. (DOCX)

S2 Table Association and dissociation constants for binding of H1N1 viruses to the 6'SLN- or 3'SLN-bound sensor surface in the presence of zanamivir. (DOCX)

S3 Table Association and dissociation constants for binding of H1N1 viruses to the 6'SLN- or 3'SLN-bound sensor surface in the absence of zanamivir. (DOCX)

## References

[1] Colman PM. Influenza virus neuraminidase: structure, antibodies, and inhibitors. Protein Sci. 1994;3: 1687–1696. doi: 10.1002/pro.5560031007 7849585 10.1002/pro.5560031007 PMC2142611

[2] Wagner R, Matrosovich M, Klenk HD. Functional balance between haemagglutinin and neuraminidase in influenza virus infections. Rev Med Virol. 2002;12: 159–166. doi: 10.1002/rmv.352 11987141 10.1002/rmv.352

[3] Xu R, Zhu X, McBride R, Nycholat CM, Yu W, Paulson JC, et al Functional balance of the hemagglutinin and neuraminidase activities accompanies the emergence of the 2009 H1N1 influenza pandemic. J Virol. 2012;86: 9221–9232. doi: 10.1128/JVI.00697-12 22718832 10.1128/JVI.00697-12 PMC3416152

[4] Mitnaul LJ, Matrosovich MN, Castrucci MR, Tuzikov AB, Bovin NV, Kobasa D, et al Balanced hemagglutinin and neuraminidase activities are critical for efficient replication of influenza A virus. J Virol. 2000;74: 6015–6020. 10846083 10.1128/jvi.74.13.6015-6020.2000 PMC112098

[5] Rudneva IA, Kovaleva VP, Varich NL, Farashyan VR, Gubareva LV, Yamnikova SS, et al Influenza A virus reassortants with surface glycoprotein genes of the avian parent viruses: effects of HA and NA gene combinations on virus aggregation. Arch Virol. 1993;133: 437–450. 8257298 10.1007/BF01313781

[6] Rudneva IA, Sklyanskaya EI, Barulina OS, Yamnikova SS, Kovaleva VP, Tsvetkova IV, et al Phenotypic expression of HA-NA combinations in human-avian influenza A virus reassortants. Arch Virol. 1996;141: 1091–1099. 8712926 10.1007/BF01718612

[7] Zanin M, Marathe B, Wong S-S, Yoon S-W, Collin E, Oshansky C, et al Pandemic swine H1N1 influenza viruses with almost undetectable neuraminidase activity are not transmitted via aerosols in ferrets and are inhibited by human mucus but not swine mucus. J Virol. 2015;89: 5935–5948. doi: 10.1128/JVI.02537-14 25810540 10.1128/JVI.02537-14 PMC4442420

[8] Hughes MT, Matrosovich MN, Rodgers ME, McGregor M, Kawaoka Y. Influenza A viruses lacking sialidase activity can undergo multiple cycles of replication in cell culture, eggs, or mice. J Virol. 2000;74: 5206–5212. 10799596 10.1128/jvi.74.11.5206-5212.2000 PMC110874

[9] Kaverin NV, Gambaryan AS, Bovin NV, Rudneva IA, Shilov AA, Khodova OM, et al Postreassortment changes in influenza A virus hemagglutinin restoring HA-NA functional match. Virology. 1998;244: 315–321. doi: 10.1006/viro.1998.9119 9601502 10.1006/viro.1998.9119

[10] Kaverin NV, Matrosovich MN, Gambaryan AS, Rudneva IA, Shilov AA, Varich NL, et al Intergenic HA-NA interactions in influenza A virus: postreassortment substitutions of charged amino acid in the hemagglutinin of different subtypes. Virus Res. 2000;66, 123–129. 10725545 10.1016/s0168-1702(99)00131-8

[11] Maines TR, Jayaraman A, Belser JA, Wadford DA, Pappas C, Zeng H, et al Transmission and pathogenesis of swine-origin 2009 A(H1N1) influenza viruses in ferrets and mice. Science. 2009;325: 484–487. doi: 10.1126/science.1177238 19574347 10.1126/science.1177238 PMC2953552

[12] Ilyushina NA, Lugovtsev VY, Samsonova AP, Sheikh FG, Bovin NV, Donnelly RP. Generation and characterization of interferon-lambda 1-resistant H1N1 influenza A viruses. PLoS One. 2017;12: e0181999 doi: 10.1371/journal.pone.0181999 28750037 10.1371/journal.pone.0181999 PMC5531537

[13] Hayden FG, Cote KM, Douglas RG. Plaque inhibition assay for drug susceptibility testing of influenza viruses. Antimictob Agents Chemother. 1980;17: 865–870. 10.1128/aac.17.5.865 PMC283889 7396473

[14] Reed LJ, Muench H. A simple method for estimating fifty percent endpoints. Am J Hyg. 1938;27: 493–497.

[15] Hoffmann E, Stech J, Guan Y, Webster RG, Perez DR. Universal primer set for the full-length amplification of all influenza A viruses. Arch Virol. 2001;146: 2275–2289. 11811679 10.1007/s007050170002

[16] Palmer DF, Dowdle WR, Coleman MT, Schild GC. Advanced laboratory techniques for influenza diagnosis. Atlanta, GA, Centers for Disease Control US Department of Health, Education, and Welfare, Immunology series. 1975;6.

[17] Wang X, Ilyushina NA, Lugovtsev VY, Bovin NV, Couzens LK, Gao J, et al Amino acids in hemagglutinin antigenic site B determine antigenic and receptor binding differences between A(H3N2)v and ancestral seasonal H3N2 influenza viruses. J Virol. 2017;91: e015120–16. 10.1128/JVI.01512-16 PMC5215349 27807224

[18] Ilyushina NA, Rudneva IA, Gambaryan AS, Bovin NV, Kaverin NV. Monoclonal antibodies differentially affect the interaction between the hemagglutinin of H9 influenza virus escape mutants and sialic receptors. Virology. 2004;329: 33–39. doi: 10.1016/j.virol.2004.08.002 15476872 10.1016/j.virol.2004.08.002

[19] Potier M, Mameli L, Belisle M, Dallaire L, Melancon SB. Fluorometric assay of neuraminidase with a sodium (4-methylumbelliferyl-alpha-D-N-acetylneuraminate)substrate. Analyt Biochem. 1979;94: 287–296. 464297 10.1016/0003-2697(79)90362-2

[20] Marathe BM, Leveque V, Klump K, Webster RG, Govorkova EA. Determination of neuraminidase kinetic constants using whole influenza virus preparations and correction for spectroscopic interference by a fluorogenic substrate. PLoS One. 2013;8: e71401 doi: 10.1371/journal.pone.0071401 23977037 10.1371/journal.pone.0071401 PMC3744557

[21] Geisler C, Jarvis DL. Effective glycoanalysis with Maackia amurensis lectins requires a clear understanding of their binding specificities. Glycobiology. 2011;21: 988–933. doi: 10.1093/glycob/cwr080 21863598 10.1093/glycob/cwr080 PMC3130539

[22] Wang W-C, Cummings RD. The immobilized leukoagglutinin from the seeds of Maackia amurensis binds with high affinity to complex-type Asn-linked oligosaccharides containing terminal sialic acid-linked α-2-3 to penultimate galactose residues. J Biol Chem. 1988;263: 4576–4585. 3350806

[23] Nicholls JM, Bourne AJ, Chen H, Guan Y, Peiris JSM. Sialic acid receptor detection in the human respiratory tract: evidence for widespread distribution of potential binding sites for human and avian influenza viruses. Respiratory Res. 2007;8: 73–83. 10.1186/1465-9921-8-73 PMC2169242 17961210

[24] Chan RWY, Chan MCW, Nicholls JM, Peiris JSM. Use of *ex vivo* and *in vitro* cultures of the human respiratory tract to study tropism and host responses of highly pathogenic avian influenza A (H5N1) and other influenza viruses. Virus Res. 2013;178: 133–145. doi: 10.1016/j.virusres.2013.03.003 23684848 10.1016/j.virusres.2013.03.003 PMC3805758

[25] Al Hajjar S, McIntosh K. The first influenza pandemic of the 21^st^ century. Ann Saudi Med. 2010;30: 1–10. 20103951 10.4103/0256-4947.59365 PMC2850175

[26] Gill JR, Sheng ZM, Ely SF, Guinee DG, Beasley MB, Suh J, et al Pulmonary pathologic findings of fatal 2009 pandemic influenza A/H1N1 viral infections. Arch Pathol Lab Med. 2010;134: 235–243. 20121613 10.5858/134.2.235 PMC2819217

[27] Lyon JB, Remigio C, Milligan T, Deline C. Acute necrotizing encephalopathy in a child with H1N1 influenza infection. Pediatr Radiol. 2010;40: 200–205. doi: 10.1007/s00247-009-1487-z 20020117 10.1007/s00247-009-1487-z

[28] Ilyushina NA, Khalenkov AM, Seiler JP, Forrest HL, Bovin NV, Marjuki H, et al Adaptation of pandemic H1N1 influenza viruses in mice. J Virol. 2010;84: 8607–8616. doi: 10.1128/JVI.00159-10 20592084 10.1128/JVI.00159-10 PMC2918990

[29] O’Donnell CD, Vogel L, Wright A, Das SR, Wrammert J, Li G-M, et al Antibody pressure by a human monoclonal antibody targeting the 2009 pandemic H1N1 virus hemagglutinin drives the emergence of a virus with increased virulence in mice. mBio. 2012;3: e00120–12. doi: 10.1128/mBio.00120-12 22647789 10.1128/mBio.00120-12 PMC3372962

[30] Ye J, Sorrell EM, Cai Y, Shao H, Xu K, Pena L, et al Variations in the hemagglutinin of the 2009 H1N1 pandemic virus: potential for strains with altered virulence phenotype? PLoS Pathogens. 2010;6: e1001145 doi: 10.1371/journal.ppat.1001145 20976194 10.1371/journal.ppat.1001145 PMC2954835

[31] Hensley SE, Das SR, Bailey AL, Schmidt LM, Hickman HD, Jayaraman A, et al Hemagglutinin receptor binding avidity drives influenza A virus antigenic drift. Science. 2009;326: 734–736. doi: 10.1126/science.1178258 19900932 10.1126/science.1178258 PMC2784927

[32] Connaris H, Govorkova EA, Ligertwood Y, Dutia BM, Yang L, Tauber S, et al Prevention of influenza by targeting host receptors using engineered proteins. Proc Natl Acad Sci USA. 2014;111: 6401–6406. doi: 10.1073/pnas.1404205111 24733924 10.1073/pnas.1404205111 PMC4035977

[33] Govorkova EA, Baranovich T, Marathe BM, Yang L, Taylor MA, Webster RG, et al Sialic acid-binding protein Sp2CBMTD protects mice against lethal challenge with emerging influenza A (H7N9) virus. Antimicrob Agent Chemother. 2015;59: 1495–1504. 10.1128/AAC.04431-14 PMC4325779 25534734

