# Supplementary figures and images for: The use of plant lectins to regulate H1N1 influenza A virus receptor binding activity
Source: PLoS One. 2018 Apr 9;13(4):e0195525. doi: 10.1371/journal.pone.0195525 (PMC5891020; doi:10.1371/journal.pone.0195525)

## Slide 1
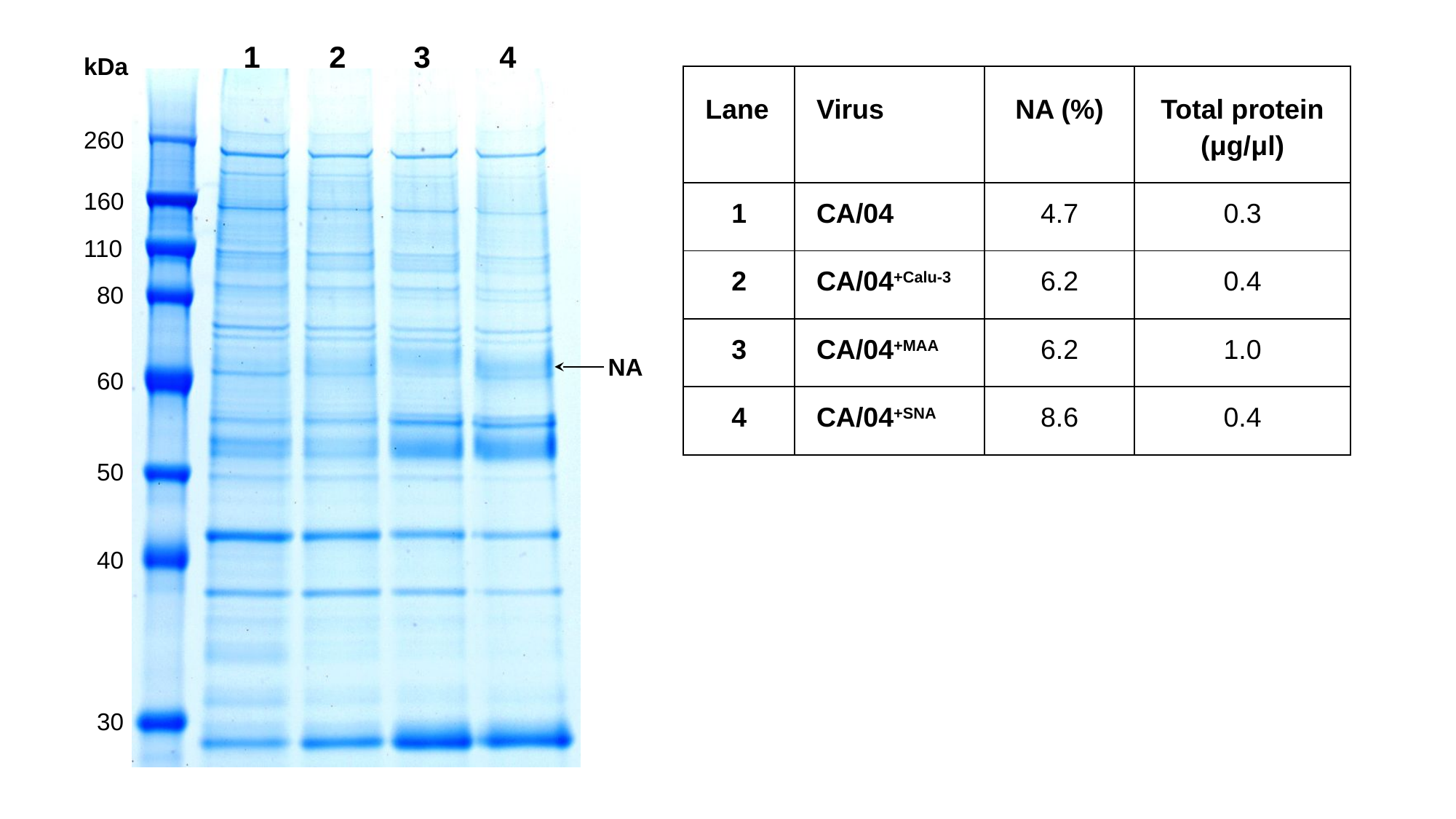

4
3
2
1
kDa
| Lane | Virus | NA (%) | Total protein (μg/μl) |
| --- | --- | --- | --- |
| 1 | CA/04 | 4.7 | 0.3 |
| 2 | CA/04+Calu-3 | 6.2 | 0.4 |
| 3 | CA/04+MAA | 6.2 | 1.0 |
| 4 | CA/04+SNA | 8.6 | 0.4 |
260
160
110
80
NA
60
50
40
30

Supplement: S1 Fig — HA and NA concentrations (%) were determined by optical densitometry and total protein content was determined by BCA protein assay. (PPTX) [file pone.0195525.s001.pptx]
