# Supplementary material for: The use of plant lectins to regulate H1N1 influenza A virus receptor binding activity
Source: PLoS One. 2018 Apr 9;13(4):e0195525. doi: 10.1371/journal.pone.0195525 (PMC5891020; doi:10.1371/journal.pone.0195525)
Supplement: S1 Table — (DOCX) [file pone.0195525.s004.docx]

**S1 Table**

Biotinylated sialylglycopolymers

| **Designation of the oligosaccharide moiety** | **Structure** |  |
| --- | --- | --- |
| SiaTn | Neu5Acα2-6GaNAcα | |
| YDS | Neu5Acα2-6Galβ1-4GlcNAc-Manα1-6  Manβ1-4GlcNAcβ1-4GlcNAc  Neu5Acα2-6Galβ1-4GlcNAc-Manα1-3 | |
| 6ʹSLN | Neu5Acα2-6Galβ1-4GlcNAc | |
| 6ʹSL | Neu5Acα2-6Galβ1-4Glc | |
| 6-Su-6ʹSLN | Neu5Acα2-6Galβ1-4(6-O-Su)GlcNAc | |
| Neu5AcBn | Neu5Acα2-OCH_2_C_6_H_4_ | |
| 6ʹSLN(Gc) | Neu5Gcα2-6Galβ1-4GlcNAc | |
| 3ʹSLN | Neu5Acα2-3Galβ1-4GlcNAc | |
