# Supplementary material for: The use of plant lectins to regulate H1N1 influenza A virus receptor binding activity
Source: PLoS One. 2018 Apr 9;13(4):e0195525. doi: 10.1371/journal.pone.0195525 (PMC5891020; doi:10.1371/journal.pone.0195525)
Supplement: S2 Table — (DOCX) [file pone.0195525.s005.docx]

**S2 Table**

Association and dissociation constants for binding of H1N1 viruses to the 6'SLN- or 3'SLN-bound sensor surface in the presence of zanamivir

|  | **k_on_ (M^-1^ x s^-1^)** | **k_off_ (1/s)** | **K_A_ (1/M)** | **K_D_ (M)** |
| --- | --- | --- | --- | --- |
| **+ 6'SLN** |  |  |  |  |
| CA/04 | 3.1 x 10^5^ | 4.4 x 10^-6^ | 7.1 x 10^10^ | 1.4 x 10^-11^ |
| CA/04^+MAA^ | 7.8 x 10^7^ | 7.8 x 10^-7^ | 1.0 x 10^14^ | 1.0 x 10^-14^ |
| CA/04^+SNA^ | 7.1 x 10^7^ | 5.2 x 10^-8^ | 1.4 x 10^15^ | 7.2 x 10^-16^ |
| CA/04^+Calu-3^ | 2.6 x 10^7^ | 1.1 x 10^-6^ | 2.4 x 10^13^ | 4.2 x 10^-14^ |
| **+ 3'SLN** |  |  |  |  |
| CA/04 | ND | ND | ND | ND |
| CA/04^+MAA^ | 3.9 x 10^5^ | 1.5 x 10^-4^ | 2.5 x 10^9^ | 4.0 x 10^-10^ |
| CA/04^+SNA^ | 3.0 x 10^7^ | 6.4 x 10^-5^ | 4.7 x 10^11^ | 2.1 x 10^-12^ |
| CA/04^+Calu-3^ | ND | ND | ND | ND |

ND – constants could not be determined due to negligible binding, which was below limit of detection.
