# Supplementary material for: The use of plant lectins to regulate H1N1 influenza A virus receptor binding activity
Source: PLoS One. 2018 Apr 9;13(4):e0195525. doi: 10.1371/journal.pone.0195525 (PMC5891020; doi:10.1371/journal.pone.0195525)
Supplement: S3 Table — (DOCX) [file pone.0195525.s006.docx]

**S3 Table**

Association and dissociation constants for binding of H1N1 viruses to the 6'SLN- or 3'SLN-bound sensor surface in the absence of zanamivir

|  | **k_on_ (M^-1^ x s^-1^)** | **k_off_ (1/s)** | **K_A_ (1/M)** | **K_D_ (M)** |
| --- | --- | --- | --- | --- |
| **+ 6'SLN** |  |  |  |  |
| CA/04 | 3.5 x 10^5^ | 9.4 x 10^-5^ | 3.8 x 10^9^ (18.7^a^) | 2.7 x 10^-10^ |
| CA/04^+MAA^ | 4.8 x 10^5^ | 1.7 x 10^-5^ | 2.9 x 10^10^ (3.5 x10^3^) | 3.5 x 10^-11^ |
| CA/04^+SNA^ | 4.2 x 10^5^ | 1.9 x 10^-4^ | 2.2 x 10^9^ (6.4 x 10^5^) | 4.5 x 10^-10^ |
| CA/04^+Calu-3^ | 3.5 x 10^5^ | 4.5 x 10^-5^ | 7.7 x 10^9^ (3.1 x10^3^) | 1.3 x 10^-10^ |
| **+ 3'SLN** |  |  |  |  |
| CA/04 | ND | ND | ND | ND |
| CA/04^+MAA^ | 5.2 x 10^5^ | 7.2 x 10^-4^ | 7.2 x 10^8^ (3.5) | 1.4 x 10^-9^ |
| CA/04^+SNA^ | 6.3 x 10^5^ | 1.3 x 10^-3^ | 5.0 x 10^8^ (940.0) | 2.0 x 10^-9^ |
| CA/04^+Calu-3^ | ND | ND | ND | ND |

^a^ – K_A_ ratio measured with inhibited NA (S2 Table) relative to uninhibited NA.

ND – constants could not be determined due to negligible binding, which was below limit of detection.
